# Supplementary material for: Structural and social factors affecting COVID-19 vaccine uptake among healthcare workers and older people in Uganda: A qualitative analysis
Source: PLOS Glob Public Health. 2024 May 29;4(5):e0002188. doi: 10.1371/journal.pgph.0002188 (PMC11135783; doi:10.1371/journal.pgph.0002188)
Supplement: S1 Text — (DOCX) [file pgph.0002188.s001.docx]

**CO-ROLL in-depth interview guide**

**Objective:** To determine the social and structural factors that influence uptake of COVID-19 vaccines in HCWs and older (>50 years) persons in Uganda

We appreciate you for accepting to take part in this interview. This interview is going to be audio recorded so we can transcribe the discussion after the meeting. Please l request that you allow me to use this recorder. Data will be anonymized and recordings deleted after the transcription.

**Topic 1 Knowledge regarding COVID – 19 vaccines**

What do you know about the COVID vaccines? (Probe for types, efficacy, use, access, side effects). What has been the main source of this information? Describe what was most useful and what was least useful in the information shared about COVID vaccines in the information documents and why?

Does this vaccine differ from other vaccines for example the hepatitis vaccine? If there are differences, please explain and describe differences or similarities

**Topic 2 Beliefs and attitudes**

What do you believe about vaccines in general (significance of vaccines in a given community?). Probe for self, family and community

In your view what are the challenges related to vaccine uptake among health workers, older people and the general community?

What in your view is the significance of receiving the COVID 19 vaccine? What do you believe about the COVID 19 vaccines?

What in your view are the barriers to vaccine uptake among the older people, professionals like health workers and the community (ask for each group)

**Topic 3 Personal experience**

You are one of the health workers/older persons who agreed to be vaccinated with the COVID -19 vaccine. What prompted you to receive the vaccine? (probe for institutional requirement, mandatory, personal choice). Please share the reasons for your taking up the vaccine. Who or which people contributed to your decision to take up a vaccine? How did they contribute to your receiving the vaccine?

What did receiving the vaccine mean to you as an individual? Did you experience side effects; please describe the side effects if you got any after the vaccination and how you got over them.

Describe any experience of fear, anxiety or any other barriers before you took the vaccine (1^st^ or 2^nd^ or both doses? what were the sources of the fears and barriers? How were you able to overcome them?

What were the reactions from your close family relations, your community/colleagues at the work place? Please share reactions from each of these groups.

If participant did not take vaccine, or chose not to go for second vaccination ***interviewer please ask*** for the reasons for this, prompt for social network/relations, personal choice or any other reasons-was it access, lack of knowledge, fear if fear from whom, what and why. Ask about rumors and beliefs (personal, community).

**Topic 4 Facilitators and barriers**

What in your view are the main facilitators to taking a covid vaccine (social, cultural, religious, legal, logistic/access factors)? Ask for self before they talk about others

What in your view are the factors that may hinder someone from taking up a covid vaccines (ask about personal, social networks, community set up, health system, logistics)? Prompts- factors of access, social institutional influence, cultural, religious, power differential, economic factor- ask for details for a lay people who do not have a health background.

What rumor’s or experiences have you heard in the community or place of work which may slow down the uptake of the covid vaccine?

**Topic 5 Future for covid vaccination**

How can individuals and the families be encouraged to take up covid vaccines? What should you do as health workers/older persons to encourage vaccine uptake among your colleagues and then among the general population?

What could be done at institutional level to increase uptake of the covid vaccine and other future vaccines that involve larger populations? (ask about type of information, communication channels, transparency of process, legal requirements, human rights-should all be vaccinated as a must)

In your view what in Uganda and other similar low and middle income countries should be done to interest people and increase uptake of the covid vaccines in order to control the virus spread?

Thank you very much for your time and contribution to this research.
