# Supplementary material for: Structural and social factors affecting COVID-19 vaccine uptake among healthcare workers and older people in Uganda: A qualitative analysis
Source: PLOS Glob Public Health. 2024 May 29;4(5):e0002188. doi: 10.1371/journal.pgph.0002188 (PMC11135783; doi:10.1371/journal.pgph.0002188)
Supplement: S2 Text — (DOCX) [file pgph.0002188.s002.docx]

**CO-ROLL Code book**

| NO | Categories | | Codes | Definitions |
| --- | --- | --- | --- | --- |
| 1. | | Knowledge | Benefits and dosage | The perceived advantages or positive outcomes associated with the COVID-19 vaccines. |
|  |  |  | Vaccine types | Information on different types of COVID-19 vaccines that they know |
|  |  |  | Side effects | Any mentioned side effects of different vaccines |
|  |  |  | Information source | Described source of information about the vaccines |
| 2 | | Beliefs and attitudes | Trust | Level of confidence in vaccines generally |
|  |  |  | Myths and misconceptions | False beliefs or misunderstandings about vaccines |
|  |  |  | Benefits of vaccination | Described benefits of COVID-19 vaccines |
| ` | | Personal Experience | Side effects | Individual experiences with vaccine-related side effects. |
|  |  |  | Fears | Personal fears or concerns related to COVID-19 vaccination |
|  |  |  | Social networks | The influence of social circles on individual attitudes and decisions related to vaccination. |
| 4 | | Barriers and facilitators | Facilitators to vaccination |  |
|  |  |  | Health education | Factors that make it easier for individuals to get vaccinated. Supportive factors, ease of access |
|  |  |  | Religious influence |  |
|  |  |  | Vaccine availability |  |
|  | |  | Barriers to vaccination |  |
|  |  |  | Myths and misconceptions | False beliefs or misunderstandings about COVID-19 vaccines held by healthcare workers and individuals aged 50 years and older in Uganda. |
|  |  |  | Absence health education | The lack or insufficiency of educational initiatives related to COVID-19 vaccination |
|  |  |  | Rumors | Unverified or unconfirmed information circulating within communities regarding COVID-19 vaccines |
|  |  |  | Vaccine access | The challenges or facilitators related to the availability and accessibility of COVID-19 vaccines. availability, accessibility, vaccine distribution. |
| 5 | | Future for COVID-19 vaccination | Policy on vaccination | Anticipation and considerations regarding future governmental regulations, guidelines, or policies related to COVID-19 vaccination. Government regulations, future policies, regulatory framework. |
|  |  |  | Access | Strategies and considerations for improving the availability and accessibility of COVID-19 vaccines in the future. Equitable access, distribution strategies, availability improvement. |
|  |  |  | Health education | Approaches for health education. Educational initiatives, awareness campaigns, |
|  |  |  | Incentives to vaccination. | Proposed motivators and rewards to encourage COVID-19 vaccination uptake. Motivational strategies, future incentives, rewards for vaccination. |
