# Supplementary material for: Structural and social factors affecting COVID-19 vaccine uptake among healthcare workers and older people in Uganda: A qualitative analysis
Source: PLOS Glob Public Health. 2024 May 29;4(5):e0002188. doi: 10.1371/journal.pgph.0002188 (PMC11135783; doi:10.1371/journal.pgph.0002188)
Supplement: S3 Text — (DOCX) [file pgph.0002188.s003.docx]

**Data - for `Structural, Social, and Contextual factors influencing COVID-19 vaccine uptake: A qualitative methods study among Healthcare Workers and Older People in Uganda’**.

1. **Knowledge regarding COVID – 19 vaccines.**

The vaccine, okay what I know, is that the vaccine was brought by the government of Uganda, to vaccinate the citizens against the pandemic, COVID-19? That is corona, yes, I know two of them. I know AstraZeneca and Sinovac. Yes, AstraZeneca is the one that was brought first, the Sinovac came in the second batch and some additional AstraZeneca. There are administered in terms of injections, in the upper left hand. That is where it is administered and uses a small needle for administering and at times, you can hardly experience any pain, in the course of the administration. Initially, there are supposed to be two doses. The first one, which one gets and then after the period of 12 weeks, one gets the second dose, and that is all, in other words, there are two doses.

What I know is that COVID-19 vaccines, it is safe and effective; the world health organization and the Uganda National Drug Authority in Uganda approved it. In that I mean like other vaccines or other drugs, it went through all the process they pass through to be a product to be used to people.

Like I cannot tell but for the vaccine to it used, it has to first be used under trails, for example animals and some people. That is why I say it pass through because they cannot bring something that they have not tried on people. So, bringing it to countries like Uganda, it means they first tested it (vaccine) in those countries, and it was effective and working for them and that is when they decided that it was good.

However, like other vaccines that we have minor side effects, but still, we expect them (the side effects) because every drug be like an injection that some minor side effects, but it doesn’t have major life-threatening side effects. Headache, severe headache, soreness on the injection site, redness, general body weakness, fevers, fatigue.

First, we use the one from serum institute of India but know we are using the one from Sweden. AstraZeneca, we give two doses, and it is 8 weeks a part and if we give someone AstraZeneca of course they expect to get those side effects and AstraZeneca, if we give it to you, you have to keep on doing those SOPs (Standard Operating Procedures) for COVID-19, you have been using because when we give you the vaccine. We are not preventing you from getting the vaccine, from getting the disease but you can contact the disease, but you must still keep on putting on your mask properly, handwashing, social distancing, avoiding crowding.

In general, I do believe that vaccines are good, and to get vaccinated is good because they (vaccines) prevent us from getting severe infection or severe diseases. They prevent from, because they help our bodies, if you are vaccinated, the body reorganizes that and can fight that specific virus when I comes to the body and fight it. Yes, and they can prevent us from getting severe diseases of those particular like if they immunize with polio, they prevent from the harsh conditions of polio like getting lame.

One thing I know, is that there are now a number of vaccines developed by different researchers may be different institutions and probably because of the nature of the disease, the way it emerged, I think they did not have the time to take vaccines through the normal processes of the vaccine development, because the routine for vaccine take some time, it goes through a number of stages but because of the nature and the onset of this pandemic. It did not give time for that to happen, though they happened to put some quality controls in. So that is what I know and probably it is a source of, and it could have contributed to myths, rumors, and speculations about the effectiveness of the vaccine probably.

One thing I know, is that there are now a number of vaccines developed by different researchers may be different institutions and probably because of the nature of the disease the way it emerged, I think they did not have the time to take vaccines through the normal processes of the vaccine development, because the routine for vaccine take some time, it goes through a number of stages but because of the nature and the onset of this pandemic, I it did not give time for that to happen, though they happened to put some quality controls with in. so that is what I know and probably it is a source of, and it could have contributed to myths, rumors, and speculations about the effectiveness of the vaccine probably

1. **Beliefs and Attitudes**

In general, the vaccines are recommended in that when we talk about the community, the populations are high and people live in congested areas, and if people are vaccinated, it will reduce the risk of spreading those diseases, as I talk now, in these, in these, as far as COVID-19 is concerned, in these western countries, people have now started watching soccer. You see.

In football fields, the condition is, you have to be vaccinated before you enter the football field or the stadium. People have now started converging in big numbers enjoying score. Which was not the case previously,

Now I think vaccines are all vaccines, there are all intended to prevent one from catching the disease and the way vaccines operate, they give you the real disease itself but in a controlled doses which helps to boost your immunity, I think it operates in the same way. It does not differ from other vaccines.

But with COVID vaccine, you still have to keep on observing the SOPS because you are likely. You can get it although the way it attacks you, may not be the same way as one who has not been vaccinated.

Being that I am somebody who is literate, I believe that vaccines in general are good to be applied because they prevent the spread of disease, especially the communicable diseases, because even COVID-19 is a communicable disease. They prevent.

I was vaccinated against polio; I was vaccinated against polio and my limbs have no problem. I was vaccinated against measles and actually, I acquired natural immunity, you see. I was vaccinated against tetanus, I even get fresh wounds without encountering any problems with them, I have never suffered from polio because I was vaccinated in my youth hood, you see, so, I look healthy because of those vaccines.

During that time, vaccines were not commonly used, so many children were dying, so vaccines, what I am saying they have reduced on medical costs because if a child is vaccinated maybe because of measles, the repercussion is not so high that it may not be even necessary to take that child to the hospital.

1. **Facilitators to COVID vaccine update**

The religious institution has a very positive attitude towards vaccination, you will find in churches, in Mosques the religious leaders are encouraging people to go for vaccination and even sensitizing them about the importance of vaccination.

Yes, the social networks have helped others to go for vaccination especially those ones who are literate. They have helped others on educating them about them importance of getting those vaccines.

The information about the vaccine, safety, effectiveness, and helping us to combat the disease, prompted me to take the vaccine. There are people from the district (district health team) who came and gave us a training prior to vaccination, so it also helped me to gain strength of coming up to be vaccinated” Female, 35 years, HCW, vaccinated.

So, when management sat, they decided to change the policy, that please if you are not vaccinated, go home and get vaccinated or if you fall sick, you pay your own bills. It was hard at first, until one of the persons; one staff got sick who was not vaccinated. Actually, she was the one campaigning to people not to be vaccinated. So, management told her “Please pay your own bills” so after that pain of paying around 8 million something. She is now the one campaigning for the vaccination. Because you know women, she did not keep quiet, so she told everyone that they made her to pay and people (staff) were like, “management is so serious like this!

At first, it was optional, we did not put much stress on everyone getting vaccinated but as time went on, we made it mandatory. I heard the RDC of Entebbe on radio when he was saying that he wants all the bodaboda to vaccinate and there are going every bodaboda stage to make sure that every bodaboda rider haves vaccination card if not, they are not going to allow them. It was today.

After the person has understood the purpose of vaccination, that person no longer has a reason not to go for vaccinate. Until management has to seat down and singled us out (non-vaccinated ones) and they actually brought one of the people who work with WHO mainly at the vaccine point to try to talk to us to remove the doubts we had.

My age prompted me and the underlying diseases I have. First, I have diabetes, I have blood pressure and on ART treatment. One of them is my age, another one is because on literacy, because I am not an illiterate person, I am literate, so I understand the importance of vaccines.

One, me as an individual, whether vaccinated or not vaccinated at any time anyone can die, so I did not see any reason why I should not go for vaccination.

I think the communication from the ministry, from the government and all other departments, if they give out effective messages, I think they can help the community to refute rumors.

Yeah, even when it came to immunizations, you have seen how the queen gets involves, how the Kabaka gets involved and that moves he communities because there are some people who say “I will only do it (vaccination)if I see an inspiration from the Kingdom. so, when they see the members of the royal family like supporting the vaccine, I think some people get motivated to participate.

Yeah, you see when we want to do an uptake of certain services, we use all those other avenues, religion plays a very bigger part. Even the influential members of the community can be relied on, once they see that so and so has gone for vaccination, then the others will be inspired.

Even if, let say if the president had not come and vaccinated, I think I will not have vaccinated, I would be wondering why he has not gone for vaccination? What is the agenda behind the vaccine, what is he fearing? He wants us to go without him going. Those would have been some of the issues that would have stopped me from going for vaccination.

1. **Barriers to COVID-19 vaccine uptake.**

That is misinformation from the community, yes, and ignorance among some members of the community. The misinformation at times comes from, in fact, some people, in fact, some of this information is politicked. There are some people who are pro the current government, and there are people who are anti the current government, you see.

But she is a medical personal, a woman between 20 and 30 years but for her she says “no my friend I can’t take this vaccine because I am longing to get pregnant” may be a three months’ time from that time we were discussing. so, there is a believe, I do not know where she gotten that believe that when you take this vaccines, it affects the chances of getting pregnant, you see. I do not know where it originated from, I do not know.

There are negative about it because some hear that not even hearing but they get it from social media that there are some people who are vaccinated, and after vaccination, they were again infected again by COVID-19. Without reliable information and that, some of them passed away but they cannot mention those that passed away. They can even give a reason that they hear that Mulogo announced that 30 people who died, why they don’t mention their names, why is that they don’t mention their places of residence or origin, they can’t mention that from Iganga or Jinja, so some say, the Baganda calls it Kiwanyi (something not true)

Don’t take the vaccine, that vaccines was made by the whites and their intentions is to get rid of us the Africans something of that kind, just as there was a myths that HIV was what, was what, I don’t know how I can put it that HIV was not real, but people were just being charmed and people took long to accept taking the ARVs, they took long. That is why when you go to Masaka, Rakia in the early 1990s you would find many orphans whereby the father and the mother perished, and you would find the children are the one taking care of themselves because there was that myths that HIV was just a charm.

I went to Kisubi hospital to be vaccinated and there are no vaccines. They gave me a telephone number to call them and check if they have the vaccines. I have called them twice and the vaccines are not yet available. Limited vaccine doses like now they come, and you tell them that the vaccines are not available, and people move several times to the hospital minus finding the vaccine.

When someone comes twice and does not get the vaccine, it will demoralize that person even not to get the vaccine. You realize that in Uganda, a few month back there were no vaccines, people wanted the vaccines but there were no vaccine.

To them, when they come and they do not have a fever, they happen to start feeling fever and severe headache after vaccination. There they conclude that they have been given poison.

May be in the beginning, being a new thing, in me I know that it attracts many people. My life maybe I am lazy; my life I do not want to struggle that maybe I would be in a long queue like many people standing in the same place. No, I want to go when, I am sure. Would not go there (hospital) because people were many at the vaccination sites.

Why do they first want us to consent? So, they mean that if I have any problem, there is nowhere I can report because I consented. Because giving a vaccine for example COVID you need to first consent. So, most of the people fear that consent form. There are questions like why are you giving us that consent form.

Barriers, that consent form, why do they want us to first consent? So, in case I have a problem now where I should go because I consented that is what people say? they have to change that questionnaire. The things that prevents people. They put like; do you have COVID-19? You are not tested. Even if you test me today and I go tomorrow for vaccination, I might get the virus after testing because I am in the hospital. Also, prevent them from taking the vaccine. That if they put free testing, at least people can come for vaccination and they explain the reason for that consent form, why they want us to first consent.

I think that is ignorance, they hear rumors that when someone get vaccinated you die. Because most people have dies, most of the people died and they were vaccinated. So, people were like “if they vaccinate you, you die” and like me we go for outreaches, they be there saying, “For us in your place we don’t have COVID-19, COVID-19 is in your place (referring to health care workers place), where can it find us” so they do not believe that COVID is there. Me, I just hear. Some may say that “me I have a relative, was vaccinated and she died”. But me I do not have a relative who died.

May be the chairperson local council, for them there are doing, they tell people to go and vaccinate and whoever completes the vaccination, comes and tells people that for them, they have completed the doses and displays the card and another one may say let me go also and vaccinate.

The radios, saying that people the vaccine are safe and effective, we for us we were vaccinated as they showed the president being vaccinated. So, people said, “if you see the president vaccinated, it means we also we shall die together with him” because you remember even him, he first rejected vaccination, that we was waiting for the Ugandan vaccine, but he then later accepted to be vaccinated.

As I told you, for example some people be there in the village and they want to be vaccinated, and they don’t have transport to the vaccination centers, remember, I told you that the vaccine is at health center threes, center fours and the hospitals. So, find like in the village, the health center nearby is health center two, and it has no vaccine. Some other people are old, may go to the hospital when there are many people at the health center and people are many. You see we are in times of COVID, but you watch on the TV when many people are conjected. Even when there were vaccinating the teachers, then were very congested, no social distancing, no wearing the mask. Now that distance, makes one think that by the time I will go to the vaccination centers, and then spend my entire day at the vaccination center, has no money to eat during the day, one my just say that I will not go because I don’t have money.

I think the rumors there are heard from different sources and the same may not be true because there are a lot of things being said, which you cannot verify. But because the ordinary person does not have the means of going out to verify whether this is true or not, they believe wholesale whatever is said. Someone may come and say that you know what; they have done this because they want you to die and leave your property and they take over your land. And the other will say, is that their agenda? Then they will not vaccinate.

Not so many rumors, apart from what they are saying that vaccines are not safe for you, they want to monitor your movements, that once you take the vaccines that it comes with unseen elements like a chip that will be used to monitor your body, those are the rumors. I think if a good number of people took the vaccine and they developed serious side effects that could hinder because on may say, so and so talk the vaccine and this happened, no I will be waiting to see, let me first hear what happens to other people.

Yeah, for example if people have to be vaccinated but the health care workers do not have gloves, then it could affect. She comes that day and there are no things to use they cannot come back tomorrow because you do not know what it took to convince that person to come for vaccination.

1. **Future of COVID-19 vaccination**

They should be sensitized about the importance of vaccination, and they should emulate those ones that have been vaccinated. they can be encouraged may be after hearing that there are some vaccination sites that have been set up by the ministry of health.

I have to encourage them by showing them my vaccination card; it bears two stamps meaning that I have received the two doses. I also have to encourage them by looking at my health to see if I have had any change as far as my health is concerned. I just ask them to look at myself and look at my card, and when I tell them that I am vaccinated, these are not my words, but evidence is here that this is my vaccination card.

What should be done, now that the budget has been read, there should be a supplementary budget to see that the government gets more funds, to buy these vaccines because these are not free there are bought? So, government should suspend may be other social services and buy vaccines. Such that almost ¾ of the people are vaccinated because the determinate factor is the availability of the vaccine.

Now the law the should take its course, should take its course whether is willing or not, the law should take its course, they should enforce it that everyone should be vaccinated if you like it or not. Of course, human rights will come in, but this is a national concern. It is a national concern and if it is a policy, it has to be enacted and the government should implement it.

First, the government has a big role to educate the people about the benefits of vaccination and the reasons why they should be vaccinated. If people get to know the importance of being vaccinated, and the importance of completing the vaccine schedules. It can help to convince people to vaccinate. Remember some people are not educated, they believe much in local medicines. So, it is the job of the government officials to educate some people. That when you take the vaccine, the vaccine will help you in this, it you will not get this. But if they do not do that

Now sensitizing, if you tell them what you are vaccinating because some are there in their homes, it is not that everyone has a radio or a TV. Now we may be saying that people are not coming for vaccination yet there are some people who want to be vaccinated but when they do not just know. but if you call a village meeting and you tell people that COVID-19 affects like this, if we don’t vaccinate our incomes are going to go down, we are in the lockdown let get vaccinated such schools are open and our children go back to school.

Also, there are village megaphones; we can get health care workers to go on those megaphones and educate the people. Remember even now gathering is not easy and you teach them the benefits of vaccination, the vaccine is like this and that, you people go and vaccinate. That is the communication I see available because now churches are closed, because for church issues it was easy because the health official just goes to the pulpit and sends the message to the people and even gatherings are not allowed.

We what I think people just lack knowledge about the vaccine and what I now see, teachers come, and they say, they told us that if you are not vaccinated, you will not be given jobs in schools. I was like, I give them consent and they said no, no Musawo just give me the injection, this turned to be a law if they want to kill us let them kill us. That is what some has, so they just need knowledge.

Me, I think if they make it mandatory. If they say that to enter a certain bank, you need to be vaccinated for COVID-19, to go the shop, one should not open a shop when not vaccinated. Each business that one is operating must indicate, now as you see that national identification is mandatory, we say the license for one to operate business must have a vaccination cards, it is the best because majority of the people want to work.

Also, empowering people with information because being ignorant can make you say a lot of things. Because that one who have never been vaccinated, is the same person who say that the vaccine does that and that, but because the person does not know the benefits of vaccination and people who suffered from COVID-19 there are the people who run for vaccination because for them they had tested the disease. But those people I the village and they had not have a test of COVID-19, they say that in their places COVID-19 cannot reach them. What do COVID-19 be looking for in the villages? now if people do have knowledge on something even if you tell them that people get vaccinated. Even if you talk like what, people will not get vaccinated, it is very hard.

I think they need first of all to believe in their health system because once you believe in the health care system then you can be encouraged but also there is need for the leadership to make an effort to convince people. Use all the different levels, for example the cultural, the religious, the political, all levels of leaders need to come in and maybe we can do a lot of sensitization. It is necessary, give all the information about it so that people can make an informed choice.

I think first, sanitization. Give all the information about it, what happens if you are not vaccinated, what happens. If you are vaccinated. I think give them all the advantages of being vaccinated and also seeing that the leadership is involved, they have already participated. When they say it will kill, and then lets us all die together, they will be motivated, knowing that for you, you are not pushing them to something that you have not taken.

I think for the general community, we have to look for the influential people that the community believe in like the area member of parliament. Talking to them and even appearing to take the vaccine in front of them and if he did not take it from that area, he/she shows them that certificate that for him/her she has taken the vaccine and it is safe. People would take the vaccine.

I think logistic management there becomes an issue; they need to improve on the management of logistics. The supply chain need to be improved, make available facilities, people support vaccination must be available and motivated but again we need to do a lot of sensitization. You cannot forget sensitization at all levels.

We can do it at all levels, through the radios, we even have community radios though I have not seen much in this area in terms of community radio you could pass on messages such that the messages pass on to everyone. You could put up some fliers for people to read and get the information, but we could also use the village health teams and whatever. Those people who move door to door could go and pass the same message as from the top.

One avail the vaccines, make sure that the vaccines are available, you remember the time when the government was pushing people to go for the vaccine and time came when people wanted to be vaccinated and the vaccines were not there. Make the vaccines available two make it to the legal requirement. Make it a policy, policy requirement that if you are not vaccinated, you do not go to school, you do not access health requirements, and people will be forced to go for vaccination. If you are not vaccinated you can’t drive a public car, a tax those public care then people will do vaccinate.

The priorities are seen by the budget allocation, increase the budget allocation but also fight graft within the health system. Corruption is the biggest problem because when we hear that they mobilized so many resources to fight COVID and then you see how much have gone to fight COVID, you get worried.
