# Supplementary material for: Structural and social factors affecting COVID-19 vaccine uptake among healthcare workers and older people in Uganda: A qualitative analysis
Source: PLOS Glob Public Health. 2024 May 29;4(5):e0002188. doi: 10.1371/journal.pgph.0002188 (PMC11135783; doi:10.1371/journal.pgph.0002188)
Supplement: Attachment — Submitted filename: Responses to the reviewer.docx [file pgph.0002188.s004.docx]

**Structural, Social, and Contextual factors influencing COVID-19 vaccine uptake: A qualitative methods study among Healthcare Workers and Older People in Uganda**

**Responses to the reviewer**

**Reviewer comment 1**

The number of people interviewed was low. Only eight of your 33 interviews were with elderly people, and they were all from the same district. Elderly people and health care workers may not have much in common. I would suggest splitting them into two populations, which may involve recruiting more elderly people. I hate to suggest to make this paper focus only on health care workers, but I am not convinced that the number and diversity of you elderly population is enough to draw some of the conclusions you draw. Taking out the elderly people and making it a paper about health care workers may focus and strengthen the manuscript.

**Authors Response**.

- In the context of COVID-19, research constantly indicated that both older persons, especially those with underlying health conditions, and healthcare workers share a heightened vulnerability to the virus. Older people are more susceptible to severe illness if infected, while Healthcare workers, due to their direct contact with infected patients on the front lines, face an elevated risk of exposure. As a result, both groups are encouraged to prioritize COVID-19 vaccinations.
- The number of older people recruited is significantly lower compared to the number of healthcare workers. However, conducting additional recruitment for older people, as recommended at this stage to match the number of healthcare workers, is extremely challenging due to the evolving context of COVID-19 and COVID-19 vaccine uptake. At the time of the initial recruitment for this study, Uganda was under the second COVID-19 lockdown, with consistent reporting of the number of COVID-19 new cases across major information platforms in the country and a high COVID-19 risk perception among the population. The current situation differs significantly from that time. The initial data from older people and the proposed additional recruitment will reflect contrasting contexts, yet context is crucial for this type of research.
- We would like to retain the data of older people in the manuscript. We mention the low number of older people recruited compared to the healthcare workers as a study weakness/limitation. With this approach, we believe we can achieve a fair balance in the manuscript since data from older person has very interesting insights in the context of the vaccine uptake.

Revised text on the study limitation are on page **26-line number 474-481.** **Revised text is below.**

**Study limitations**

A notable limitation of this study pertains to the relatively low number older persons included in the study sample compared to the HCWs. Out of a total of 33 interviews conducted, only eight involved older people and 25 for HCWs. Furthermore, older persons interviews were exclusively sourced from a single district, Wakiso district, a mostly semi-urban/urban setting, the study results may not comprehensively reflect the lived experiences of rural older persons in relation to COVID-19 vaccine uptake. Future research should strive to include a more extensive and varied older persons participant group to enhance the comprehensiveness of the results.

**Reviewer comment 2.**

Please go into greater detail in the methods section on how you selected the number of people you chose to survey.

**Authors Response.**

- Thank you for the suggestion. We have included more detailed information in the methods section regarding how we selected the number of people for the survey. Specific changes to this section are on page 7 of the manuscript, line numbers between **124-141 of page 7.** **The revised text is below;**

**Study participants, and sampling.**

Using the participant identification logs of the COVID-19 vaccine acceptability and immunogenicity study, we conducted a purposive sampling process to identify and invite 33 individuals who had been enrolled in the preceding three weeks to participate in the in-depth interviews.

We aimed to aimed to capture a wide range of perspectives by purposively including participants with diverse demographics. Selection was based on sex, occupation, education, cadre of the HCWs (Doctors, Nurses, Laboratory technicians, hospital support staff and administrators). We also ensured that our sample included participants with varying vaccination status. This was important to explore the perceptions and experiences of those who had been vaccinated and those who had not.

To account for potential variations in vaccine acceptability among different healthcare settings, we selected participants from three distinct hospitals: Kisubi hospital (n=8), Entebbe Regional Referral hospital (n=8), and Villa Maria hospital (n=9). Additionally, we included 8 older participants from WOPs. This approach allowed us to move beyond solely studying vaccine acceptability among HCWs and explore the perspectives of a more diverse sample within a COVID-19 at-risk population. The combination of these factors guided our selection process, ensuring that we had a diverse group of participants for the in-depth interviews.

**Reviewer comment 3.**

Please discuss more in the methods section how you performed your results coding and how many people verified the conclusions from each interview.

**Authors Response**

- We have provided additional information in the methods section to offer a description of the coding process. Revised text is on Pages 8-9. Line number 160-169.

**The revised text is below:**

**Data management and analysis.**

Anonymized audio files were transferred onto encrypted password-protected computers, transcriptions and translations were done by the RAs, and anonymized transcripts were transferred to a secure data server at the Medical Research Council /Uganda Virus Research Institute and London School of Hygiene and Tropical Medicine (MRC/UVRI and LSHTM) Uganda Research Unit.

Data analysis process involved an iterative approach with two research assistants, under the guidance of a senior social scientist. The research team collaboratively reviewed and discussed transcripts to identify and reach a consensus on common codes. A codebook was developed based on a priori data categorization and emergent categories that came from the coding process. The codebook included definitions and examples for each code to facilitate consistent coding.

To ensure consistency and reliability of the coding process, two research assistants independently coded a subset of transcripts, compared results and any differences in coding were resolved through discussion led by the senior social scientist until a consensus was reached. Patterns from the data led to the themes identified in this paper informed by the layers from the SEM.

**End.**
