# Supplementary material for: Structural and social factors affecting COVID-19 vaccine uptake among healthcare workers and older people in Uganda: A qualitative analysis
Source: PLOS Glob Public Health. 2024 May 29;4(5):e0002188. doi: 10.1371/journal.pgph.0002188 (PMC11135783; doi:10.1371/journal.pgph.0002188)
Supplement: Attachment — Submitted filename: Response to the reviewer.docx [file pgph.0002188.s005.docx]

Structural, Social, and Contextual factors influencing COVID-19 vaccine uptake: A qualitative methods study among Healthcare Workers and Older People in Uganda

Responses to the reviewers (Reviewer 2)

**Title**

**Reviewer’s comment**

The title needs minor but very important syntax modifications. This study, in no way can measure or infer the level of “influence” going by the study design and methodological approaches used. Some of the constructs mentioned in the title for measurement, particularly contextual factors, appear vague and redundant. A more appropriate title suggested would be; “Structural and Social factors involved with COVID-19 vaccine uptake among Healthcare Workers and Older People in Uganda: A qualitative analysis”

**Response**

Thank you for suggestions.

We have revised the title to better reflect the content of our study:

“Structural and Social factors affecting COVID-19 vaccine uptake among Healthcare Workers and older people in Uganda: A Qualitative Analysis.”

**Abstract**

**Reviewer’s comment**

Abstract: The objective of the study as stated in the abstract needs to be appropriately stated to adequately align with the problem phenomenon warranting the study which has not been well stated. Methodology needs editorial revising to establish clarity.

**Responses**

Thank you – we have revised the abstract in line with your suggestions, while also ensuring that we remain within the word limit of 300 words.

**Background/introduction**

**Reviewer’s comment**

The background has not operationalized the principles extant in the title and objective of the study. The coherence of the thesis of the study is weak and has not established any argument needing to demonstrate proof of concept warranting data collection and analysis. The statement in Lines 56 - 59 is redundant and has not contributed to the context of the narrative.

There are some grammatical issues to be treated. Line 128. Line 55: “had caused 12.2 million confirmed cases (256,542 deaths) in Africa and 170,544 confirmed cases (3,632 deaths) in Uganda…” should read; “...had caused 12.2 million confirmed cases with 256,542 deaths in Africa and 170,544 confirmed cases with 3,632 deaths in Uganda…”.

There are some grammatical issues to be treated. Line 128. Line 55: “had caused 12.2 million confirmed cases (256,542 deaths) in Africa and 170,544 confirmed cases (3,632 deaths) in Uganda…” should read; “...had caused 12.2 million confirmed cases with 256,542 deaths in Africa and 170,544 confirmed cases with 3,632 deaths in Uganda…”

**Response**

Thank you for your suggestions we have checked through this section and made changes in line with your suggestions.

**Fallout from an analysis of the background narrative**:

a. What is the problem and argument the study seeks to establish? This is not succinctly apparent in the background narrative. At least, from lines 54-69 should have appropriately introduced the basis of delayed vaccine rollout, efficacy expected and likely problems to be encountered with such modality, considering global antecedents with vaccine acceptance challenges with other vaccines already in use for different communicable diseases before providing citations of study demonstrating acceptance challenges with the vaccines for Covid-19 as in lines 64, 70-79. Introducing the statement “Structural, social, and contextual factors may contribute to vaccine confidence and affect the uptake of the COVID-19 vaccine among HCWs and older people in Uganda” in line 80 has not contributed appropriately to coherence and clarity of the study argument. What is meant by structural factors, or social factors or contextual factors?

b. By stating “We set out to explore the structural, social, and contextual factors that influence the uptake of COVID-19 vaccines among HCWs, and older people aged 50 years using the Social Ecological Model (SEM) to structure our analysis” introduces a confusion of whether a statement of objective is intended or introduction of the theoretical framework.

c. The theoretical framework: Theories and models are very important tools in public health and health promotion research because they offer the basis for understanding individual behaviour and those factors that are associated with such behaviour. McLeroy et al (1987) clarified the importance of models within the context of an ecological perspective stated that behaviour results from the interaction between the individual and the environmental determinants, such as biological, psychological, socio-cultural and structural spheres which facilitates the behavioural outcomes. The choice of the Social Ecological Model (SEM) in this study appears appropriate. However, it must be noted that it is a framework that provides only context of levels of relationships and interactions but does not provide constructs to be measured and therefore, requires further analysis of the dynamics involved at the various levels to appropriately apply SEM to operationalize the dynamics involved at each level of the context. Any application of SEM without recourse to this micro-level consideration would easily be deemed frivolous and flawed. In the statement of lines 93-95; “Using this framework, we explored participants' knowledge, beliefs, and attitudes about COVID-19, personal experience about COVID-19 vaccines, facilitators and barriers to vaccine uptake, and opinions on the future of vaccine rollout” raises the questions of on what scientific basis has the constructs of knowledge, beliefs, and attitudes about COVID-19 been derived? Kindly review the theoretical framework and re-define the applications of SEM to operational sources of the constructs at the intra-personal, inter-personal, community and policy levels. You may consider the health belief model at the intra-, social learning at the inter- and probably social marketing or adoption of innovation at the community-, and policy levels. There are variables/constructs fitting these specified models to adequately align the thesis of the study with regards to figure 2 representations and the methodology.

**Response**

Thank you very much for your comments and observations. We have edited the paper to make adjust the description of the framework which as we think you are suggesting in your comments – was more of a tool for ordering our material, than deeper analysis. We believe the SEM serves this purpose well but pointing to the different areas of influence on vaccine update. We do not consider our approach to be either `frivolous’ or `flawed’ given we are not claiming to make any theoretical advancement. Indeed, our purpose – as we state -- is to `describe the social and structural factors affecting the uptake of COVID-19 vaccines’. Given two other reviewers did not take issue with our approach, we respectfully beg to differ with you on our use of SEM.

4. The results: The exclusively qualitative approach has constituted a weakness in the appropriate application of the framework to adequately answer the emerging research questions in the study. I would have thought that the qualitative aspect of the study design to address context-relevance and quantitatively establish the proof of associations between structural, social and the outcome variable of covid-19 vaccine uptake.

**Response**

Yes, a quantitative methods study might address the topic rather differently. However, we did not conduct a quantitative methods study – we conducted a study using qualitative methods which has allowed us to describe the views and perceptions of our target populations on the COVID-19 vaccine roll-out. We do not consider this to be a limitation – it is a different type of study from one using quantitative methods.
